# Phase I clinical trial of adoptive cellular immunotherapy with APN401 in patients with solid tumors

**DOI:** 10.1186/2051-1426-3-S2-P175

**Published:** 2015-11-04

**Authors:** Pierre Triozzi, Mitra Kooshki, Angela Alistar, Rhonda Bitting, Amy Neal, Guenther Lametschwandtner, Hans Loibner

**Affiliations:** 1 Wake Forest University, Winston-Salem, NC, USA; 2 Apeiron Biologics, Vienna, Austria

## Background

Casitas-B-lineage lymphoma protein-b (Cbl-b), an E3 ubiquitin ligase, has been identified as a key intracellular checkpoint limiting lymphocyte activation. Inhibiting Cbl-b has been shown to enhance T cell and natural killer cell mediated antitumor activity in mouse tumor models. APN401 is a suspension of autologous peripheral blood mononuclear cells (PBMCs) transfected with a siRNA that knocks down Cbl-b. A Phase I clinical trial has been initiated to establish feasibility and to determine toxicity of the intravenous infusion of APN401.

## Methods

Patients with metastatic solid tumors no longer responding to standard therapies have been enrolled into one of three successive dosing cohorts in which they received a single intravenous infusion of 5, 10, or 50 x10^5^/kg transfected PBMCs. Eligibility criteria included at least 4 weeks since prior therapy, ECOG performance status 0-1, and adequate hematologic and organ function. Patients with active autoimmune disease or a requirement for immune suppressive drugs were excluded. PBMCs were collected by leukapheresis. The following day PBMCs were transfected with Cbl-b siRNA ex vivo by electroporation and then infused over 30 minutes.

## Results

Three patients have been treated in each of the three dosing cohorts. PBMCs were successfully collected and transfected with Cbl-b siRNA in all patients, which included 6 with pancreatic, 2 with colon, and 1 with kidney cancers. Among PBMCs, CD56 cells were most efficiently transfected (55%), followed by CD3 (46%), CD19 (45%), and CD14 (23%) cells. Cbl-b-siRNA-transfected PBMCs produced 4-fold more interferon gamma and 2-fold more interleukin-2 in response to stimulation with anti-CD3/CD28 antibody in vitro. APN401 infusions were well tolerated. One patient in the first, three in the second, and two in the third cohort developed grade 2 chills at the completion of the infusion. These responded to meperidine. Grade 3 or 4 toxicities were not observed. No immediate hypersensitivity was noted. There was no evidence of autoimmune adverse effects. Patients are being followed for systemic immune effects.

## Conclusion

A single intravenous infusion of 50 x10^5^/kg of APN401, autologous Cbl-b silenced PBMCs, into patients with refractory solid tumors is feasible and safe. The results support Phase II clinical trials of multiple infusions of APN401.

